# Differentially Coexpressed Disease Gene Identification Based on Gene Coexpression Network

**DOI:** 10.1155/2016/3962761

**Published:** 2016-11-30

**Authors:** Xue Jiang, Han Zhang, Xiongwen Quan

**Affiliations:** College of Computer and Control Engineering, Nankai University, Tianjin 300350, China

## Abstract

Screening disease-related genes by analyzing gene expression data has become a popular theme. Traditional disease-related gene selection methods always focus on identifying differentially expressed gene between case samples and a control group. These traditional methods may not fully consider the changes of interactions between genes at different cell states and the dynamic processes of gene expression levels during the disease progression. However, in order to understand the mechanism of disease, it is important to explore the dynamic changes of interactions between genes in biological networks at different cell states. In this study, we designed a novel framework to identify disease-related genes and developed a differentially coexpressed disease-related gene identification method based on gene coexpression network (DCGN) to screen differentially coexpressed genes. We firstly constructed phase-specific gene coexpression network using time-series gene expression data and defined the conception of differential coexpression of genes in coexpression network. Then, we designed two metrics to measure the value of gene differential coexpression according to the change of local topological structures between different phase-specific networks. Finally, we conducted meta-analysis of gene differential coexpression based on the rank-product method. Experimental results demonstrated the feasibility and effectiveness of DCGN and the superior performance of DCGN over other popular disease-related gene selection methods through real-world gene expression data sets.

## 1. Introduction

High throughput biotechnologies have been routinely used in biological and biomedical researches. As a result, tremendous amounts of large-scale omics data have been generated, providing not only great opportunities but also challenges for understanding the molecular mechanism of complex diseases. Screening disease-related genes by analyzing gene expression data represents one of these opportunities and challenges.

Differentially expressed gene analysis represents one of the most fundamental methods for disease-related gene identification by using gene expression data. Differentially expressed gene analysis methods select the genes which give the greatest contribution to diseases classification by comparing the changes of gene expression levels between normal samples and disease samples [1]. Those selected differentially expressed genes are considered as candidates to play a pathogenic role, termed disease-related genes or disease genes. The papers [2–4] firstly conducted gene expression analysis using statistical test, then ranked the genes in descending order according to the statistics which define the degree of gene differential expression, and finally selected the top genes as disease genes. The papers [5, 6] reconstructed gene expression data using nonnegative matrix factorization and conducted analysis of differentially expressed genes according to the new constructed matrix. The papers [7, 8] selected differentially expressed disease-related genes by minimizing the prediction error of classification. The papers [9, 10] obtained different disease-related gene subsets by using different samples and then got the optimal disease-related gene subset by integrating multiple disease-related gene subsets. This strategy in [9, 10] improved the correctness and robustness of disease-related genes. Though differential expression genes have high correlation with disease phenotypes and diseases classification, these methods may not fully consider the changes of interactions between genes in different cell states and the dynamic processes of gene expression levels during disease development and progression for disease gene selection [11]. It is reported that complex diseases are often related to the changes of interactions between genes. Thus, some disease-related genes may not be identified by only finding differentially expressed genes.

Differentially coexpressed genes (DCG) analysis is different from the individual differentially expressed gene analysis methods. Differentially coexpressed genes are highly correlated under one cell state but uncorrelated under another cell state [12, 13]. Since the normal functions of genes are destroyed in disease cell state, the coexpression patterns in normal cell state are broken down [14]. Differential coexpression gene identification is very helpful for discovering potential biomarkers and understanding the pathophysiology of complex disease. The existing methods for identifying differentially coexpressed genes focused on gene-gene coexpression analysis or gene coexpression modules analysis. The earliest related research [15] proposed an additive model and a stochastic search algorithm to investigate differentially coexpressed genes. The paper [16] selected pairs of differentially coexpressed genes using a statistical method. The paper [17] constructed gene network by measuring the correlation between genes using mutual information and conducted clique analysis to get the differentially coexpressed genes.

As the normal interactions between genes would be greatly affected by abnormal protein in neurodegenerative diseases, such as Huntington disease, the symptoms of the disease grow progressively more severe and are debilitated with time, eventually leading to death. The disease gene (IT15) of Huntington disease which produces the abnormal disease protein (Htt) has already been discovered [18]. However, there is still no cure for this disease. In fact, the exact pathogenesis of Huntington disease has not yet been illustrated completely. The changes of interactions between genes caused by the abnormal protein are reflected as the changes of gene expression level. It is well known that the similar expression patterns represent the same biological process or function [19–21]. The changes of interactions between genes can be reflected by the changes of expression patterns in coexpression network, as gene coexpression network is constructed by using gene expression data. Thus, we can identify the differentially coexpressed disease-related genes by studying and analyzing the dynamic changes of gene coexpression patterns in phase-specific gene coexpression networks. This is of great significance to understand the pathogenesis of neurodegenerative diseases.

In this study, we developed a differentially coexpressed disease gene identification method based on gene coexpression network (DCGN) for identifying differential coexpression disease-related genes. We firstly constructed a series of phase-specific gene coexpression networks using gene expression data of different time points and defined the conception of differential coexpression of genes in coexpression network. Then, we designed two metrics to measure the value of gene differential coexpression according to the change of local topological structures between different phase-specific coexpression networks. Finally, we conducted meta-analysis of gene differential coexpression according to the rank-product method [22]. This paper provided a novel framework and a method to evaluate the value of differential coexpression for each gene rather than gene pairs or genes modules. Experimental results demonstrated the feasibility and effectiveness of DCGN and the superior performance of DCGN over other popular disease-related gene selection methods through real gene expression data sets.

The rest of this study was organized as follows: the DCGN was presented in Section 2. Experiments that demonstrated the performance of DCGN were reported in Section 3. The overall discussion with some suggestions for future research was presented in the last section.

## 2. Method

**Figure 1:**
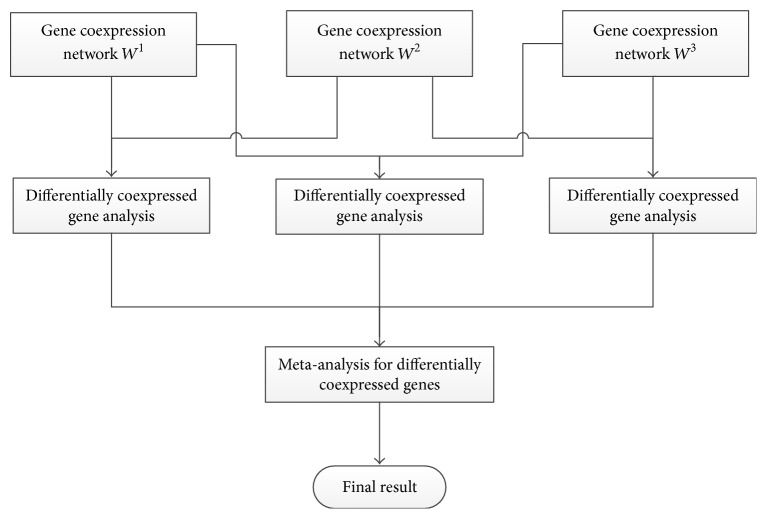
An overview of the novel framework for differentially coexpressed disease gene identification.

In this section, we firstly presented the overview of the novel framework for differentially coexpressed disease gene identification. The framework was shown in Figure 1.

Next, the gene coexpression network was introduced and the construction of gene coexpression network by using WGCNA software package [23, 24] was briefly described. Then, the conception of gene differential coexpression in coexpression network was defined and two metrics were proposed to measure the value of gene differential coexpression according to the change of local topological structures between phase-specific networks. Finally, the meta-analysis of gene differential coexpression based on the rank-product method was described.

### 2.1. Gene Coexpression Network

The gene coexpression network is usually constructed by measuring the gene expression similarity, which represents the coexpression relationships between genes [25]. Each node in the network represents a single gene. Each edge connecting two genes indicates the coexpression.

Let *X* ^*t*^ = [*x*_*ijt*_] ∈ *R* ^*n*×*m*^ denote gene expression data in *t*-phase. *x*_*ijt*_ represents expression level of gene *i* in sample *j* at *t*-phase. *n* and *m* denote the number of genes and number of samples, respectively.

In order to study the dynamic changes of interactions between genes, we firstly constructed phase-specific gene coexpression network by using the WGCNA software package [23, 24], ensuring that the network is scale-free [26]. In the coexpression network *G* = (*V*, *E*), *V* is the set of nodes, where one node corresponds to a gene. *E* is the set of edges, showing the mutual interactions between genes. *w*_*ij*_ is the weight of the edge connecting nodes *i* and *j*, *w*_*ij*_ ∈ (0,1). It should be noted that the stronger the Pearson correlation is, the larger the weight is. *W* = [*w*_*ij*_] is the weight matrix of gene coexpression network. The adjacency matrix is *A* = [*a*_*ij*_], where *a*_*ij*_ represents the interactions between nodes *i* and *j*. The calculation of *a*_*ij*_ is given by

(1) $$\begin{array}{c} a_{ij}=\left\{\begin{array}{cc} 1, & \text{if }w_{ij}\neq 0;\\ 0, & \text{else}. \end{array}\right. \end{array}$$

The transition matrix is *M* = [*m*_*ij*_], where *m*_*ij*_ denotes the probability of transition from node *i* to node *j*. The calculation of *m*_*ij*_ is given by

(2) $$\begin{array}{c} m_{ij}=\left\{\begin{array}{cc} \frac{w_{ij}}{\sum_{j\in N_{i}}^{\;}w_{ij}}, & \text{if }N_{i}\neq ⌀;\\ 0, & \text{else}. \end{array}\right. \end{array}$$

Here, *N*_*i*_ is the set of neighboring nodes of *i* in gene coexpression network *W*.

### 2.2. Gene Differential Coexpression Analysis

In this subsection, according to the change of local topological structures between different phase-specific gene coexpression networks, the gene differential coexpression analysis was conducted. The conception of gene differential coexpression was defined and two metrics were proposed to measure the value of gene differential coexpression.

**Definition 1 .**

Gene differential coexpression: in gene coexpression network *W* ^*t*^, *SW*_*i*_^*t*,*k*^ represents the *i*-centric system, a subnet including gene *i* and its *k*-level neighboring nodes (nodes that can be reached within *k* steps from node *i*). *W* ^*t*_1_^ and *W* ^*t*_2_^ denote the gene coexpression network in *t*_1_-phase and *t*_2_-phase, respectively. The differential coexpression of gene *i* represents the change of topological structures between *SW*_*i*_^*t*_1_,*k*^ and *SW*_*i*_^*t*_2_,*k*^.

In this paper, we designed two metrics to measure the value of gene differential coexpression. The first one is to evaluate the value of gene differential coexpression based on the local topological structures similarity. The second one is to evaluate the value of gene differential coexpression based on the variation of local topological information.

*(1) The Value of Gene Differential Coexpression Based on the Local Topological Structures Similarity*. In this subsection, we firstly defined the conception of the value of gene differential coexpression based on the local topological structures similarity between two different phase-specific gene coexpression networks.

**Definition 2 .**

The value of differential coexpression of gene *i* based on the local topological structures similarity between coexpression networks *W* ^*t*_1_^ and *W* ^*t*_2_^ is the topological similarity between *SW*_*i*_^*t*_1_,*k*^ and *SW*_*i*_^*t*_2_,*k*^. The value can be calculated according to the following equation:

(3) $$\begin{array}{c} d_{i}^{t_{1}t_{2},k}=1-\frac{\left|N_{i}^{t_{1},k}\cap N_{i}^{t_{2},k}\right|}{\left|N_{i}^{t_{1},k}\cup N_{i}^{t_{2},k}\right|}. \end{array}$$

Here, *N*_*i*_^*t*,*k*^ is the set of connections between genes in *SW*_*i*_^*t*,*k*^.

*(2) The Value of Gene Differential Coexpression Based on the Variation of Local Topological Information*. In this subsection, the information of an edge was firstly described. Then, the conception of the value of gene differential coexpression based on the variation of local topological information between two different phase-specific gene coexpression networks was proposed.

In gene coexpression network *W* ^*t*^, we designed a function (shown as (6)) to evaluate the information of an edge. Then, according to (7), the value of gene differential coexpression based on the variation of local topological information can be calculated.

**Definition 3 .**

The value of differential coexpression of gene *i* based on the variation of local topological information between coexpression networks *W* ^*t*_1_^ and *W* ^*t*_2_^ is the total variation of topological information caused by the topological structures differences between *SW*_*i*_^*t*_1_,*k*^ and *SW*_*i*_^*t*_2_,*k*^. The value can be calculated according to (7).

The details of computing the value of gene differential coexpression based on the variation of local topological information are shown below.

**Step 1 .**

In gene coexpression network *W* ^*t*^, extract submatrix *P* ^*t*^ = [*p*_*ij*_^*t*^], which denotes the subnetwork *SW*_*i*_^*t*,*k*^, from the transition matrix *M* ^*t*^, *j* ∈ *N*_*i*_^*tk*^. Here, *N*_*i*_^*tk*^ is the set of genes *i* and their *k*-level neighboring nodes in gene coexpression network *W* ^*t*^.

**Step 2 .**

In subnetwork *SW*_*i*_^*t*,*k*^, calculate the maximum probability of transition from node *i* to node *j* with least steps. We use *p*_*ij*_^max^ to denote the maximum transition probability, *j* ≠ *i*, *j* ∈ *N*_*i*_^*tk*^.

**Step 3 .**

Normalize the probability of transition from node *i* to node *j*. The normalized probability of transition from nodes *i* to *j* is calculated by

(4) $$\begin{array}{c} p_{ij}=\frac{p_{ij}^{max}}{\sum_{j\in N_{i}^{tk},j\neq i}^{\;}p_{ij}^{max}},\;j\in N_{i}^{tk}. \end{array}$$

After the above three steps, according to the topological information of *SW*_*i*_^*t*,*k*^, we transformed the *i*-centric subnetwork *SW*_*i*_^*t*,*k*^ into a network *DW*_*i*_^*t*,*k*^. In *DW*_*i*_^*t*,*k*^, node *i* connects to node *j* directly with the transition probability *p*_*ij*_, *j* ∈ *N*_*i*_^*tk*^. It needs to be noted that, in the *i*-centric network *DW*_*i*_^*t*,*k*^, there are no connections between other nodes. To get the value of gene differential coexpression, we still need to do the following steps.

**Step 4 .**

To ensure that the strong coexpressed interactions between genes carry larger amount of information, we need to modify *p*_*ij*_ as

(5) $$\begin{array}{c} p_{ij}^{t,k}=\frac{1/p_{ij}}{\sum_{j\in N_{i}^{tk},j\neq i}1/p_{ij}},\;j\in N_{i}^{tk}. \end{array}$$

**Step 5 .**

In *i*-centric subnetwork *SW*_*i*_^*t*,*k*^, the information that represents the connection between node *i* and node *j* is *I*_*ij*_^*t*,*k*^. The calculation of *I*_*ij*_^*t*,*k*^ is given by

(6) $$\begin{array}{c} I_{ij}^{t,k}=-\frac{\ln\left(p_{ij}^{t,k}\right)}{p_{ij}^{t,k}},\;j\in N_{i}^{tk}. \end{array}$$

The value of differential coexpression of gene *i* based on the variation of topological information between coexpression networks *W* ^*t*_1_^ and *W* ^*t*_2_^ is calculated by

(7) $$\begin{array}{c} I_{i}^{t_{1},t_{2}}=\underset{j\in N_{i}^{t_{1}k}-N_{i}^{t_{2}k}}{\sum }I_{ij}^{t_{1},k}+\underset{j\in N_{i}^{t_{2}k}-N_{i}^{t_{1}k}}{\sum }I_{ij}^{t_{2},k}. \end{array}$$

The variation of topological information can be also interpreted as the total information change when one topological structure is replaced by another topological structure.

### 2.3. Meta-Analysis of Gene Differential Coexpression

After getting the value of gene differential coexpression according to any two different phase-specific coexpression networks, we ranked the genes in descending order according to the value of gene differential coexpression. *r*_*i*_^*t*_1_,*t*_2_^ denotes the ranking of gene *i* based on the coexpression network *W* ^*t*_1_^ and *W* ^*t*_2_^. It needs to be noted that the larger the value of gene differential coexpression is, the higher the ranking of gene is. That means high ranking gene is of large probability of being disease-related gene. According to the rank-product method [22], the comprehensive ranking of gene *i* is

(8) $$\begin{array}{c} R_{i}={\left(\;\prod_{t_{1},t_{2}\in T,t_{1}\neq t_{2}}r_{i}^{t_{1}t_{2}}\;\right)}^{\left(1/C\right)}. \end{array}$$

Here, *C* = *N*(*N* − 1)/2, where *N* is the number of coexpression networks. Then, rank the *R*_*i*_, *i* ∈ *V*, in ascending order to get the final rank list of genes. It is important to note that the higher the ranking of gene is, the larger the probability of differentially coexpressed disease-related gene is.

## 3. Experimental Results

In this section, experiments were conducted to verify the feasibility of the novel framework for disease gene identification and the effectiveness of DCGN proposed in this paper. Two time-series real data sets were used in our study, one is of Huntington disease (HD) and the other one is of type 2 diabetes mellitus (T2DM). We firstly described the analysis process by using gene expression data of HD in detail. Then, the results in the gene expression data sets of T2DM were analyzed. Compared with other statistical disease gene selection methods, the superior performance of DCGN was illustrated. Finally, to explore the characters of DCGN based on different measures, a case study was conducted.

### 3.1. Gene Expression Data of HD

The gene expression data of HD used in our study was RNA-seq data from http://www.hdinhd.org/. It was obtained from striatum tissue of Huntington disease mice. Huntington disease is one kind of neurodegenerative diseases. It is due to a triplet repeat elongation in the Huntington gene (IT15), which leads to neuronal malfunction and degeneration through a large scale of different interactions between genes and a number of different molecular pathways. The symptoms of the disease grow progressively more sever and are debilitated with time, eventually leading to death.

In the gene expression data, there are 4 genotypes, including polyQ 92, polyQ 111, polyQ 140, and polyQ 175. Each genotype has 8 replications. Thus, the gene expression data has 32 samples totally. According to the age of experimental mouse, there are 3 gene expression data sets in different phases, including gene expression data of 2-month-old HD mouse, gene expression data of 6-month-old HD mouse, and gene expression data of 10-month-old HD mouse. In order to clearly demonstrate the information of the experimental data, Table 1 was carried out. In order to filter out noise genes, we conducted a preprocessing step and selected 8815 genes from the total 23351 genes in the gene expression data. The data of modifier genes were from [27], which contained 520 genes in training set, including 89 disease genes and 431 nondisease genes.

### 3.2. The Topological Information of Gene Coexpression Network

The gene coexpression network was constructed by using the WGCNA software package [23, 24]. *W* ^*t*^ denotes the gene coexpression network constructed with gene expression data of *t*-month-old HD mouse. The topological information of the three phase-specific networks is shown in Table 2. As shown in Table 2, there exist big differences between the topological structures of the three gene coexpression networks though we used the same standard to construct these networks.

To illustrate differences of the three networks, we analyzed the distribution of degrees, weighted degrees, and weights in each gene coexpression network.

Investigating the similarity between different coexpression networks, we can know that the similarity between *W* ^2^ and *W* ^6^ is only 0.032, the similarity between *W* ^2^ and *W* ^10^ is 0.042, and the similarity between *W* ^6^ and *W* ^10^ is 0.111.

**Figure 2:**
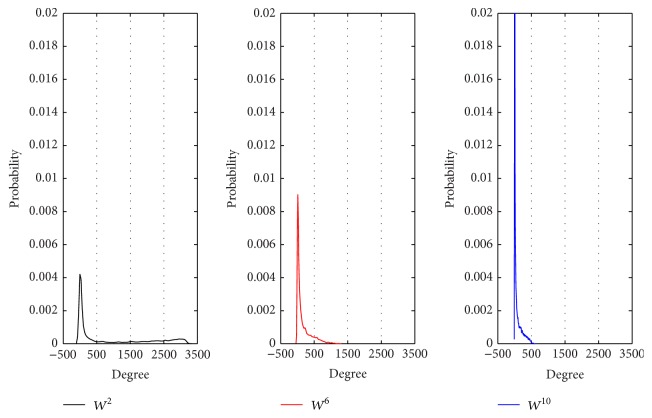
The distribution of degrees in *W* ^2^, *W* ^6^, and *W* ^10^.

**Figure 3:**
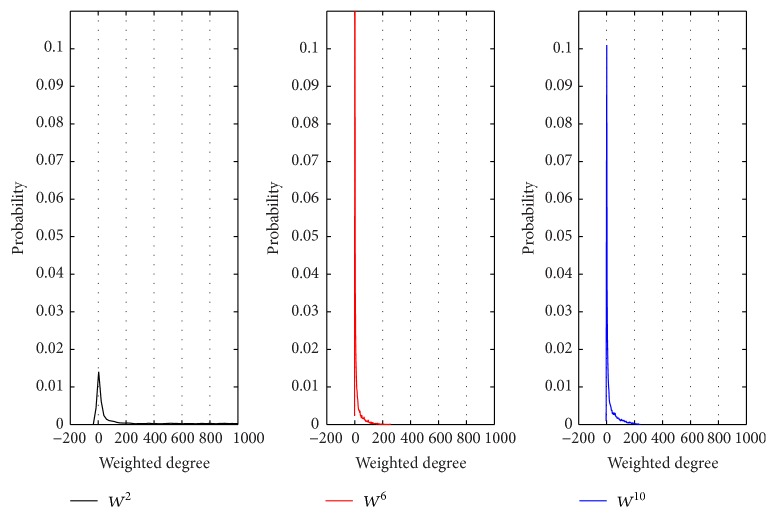
The distribution of weighted degrees in *W* ^2^, *W* ^6^, and *W* ^10^.

**Figure 4:**
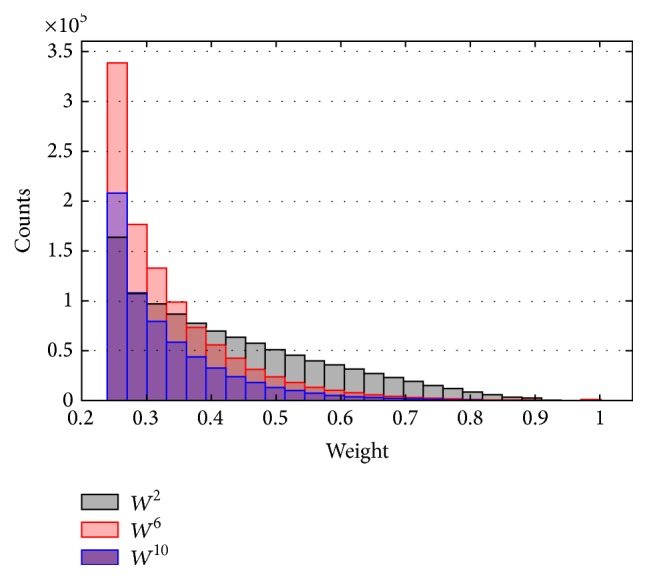
The distribution of weights in *W* ^2^, *W* ^6^, and *W* ^10^.

From Figures 2, 3, and 4, we can get the following information and conclusions. Firstly, for *W* ^2^, there are denser connections (Figures 2 and 3) and the degrees of hub nodes in *W* ^2^ are about 3000 while most nodes have large degrees (Figure 2). At the same time, the connections between genes are also stronger (Figure 4). The above topological information of *W* ^2^ suggests that the interactions between genes are very active in 2-month-old Huntington disease mouse. Secondly, for *W* ^6^, compared with *W* ^2^, *W* ^6^ has quite sparse connections (Figures 2 and 3) and the degrees of hub nodes in *W* ^6^ are about 800 while only few nodes have large degrees (Figure 2). Moreover, the most connections between genes are not strong (Figure 4). This topological information of *W* ^6^ suggests that the interactions between genes in 6-month-old Huntington disease mouse are greatly changed. Thirdly, for *W* ^10^, the connections in *W* ^10^ are sparser and weaker (Figures 2, 3, and 4), indicating that the interactions between genes in 10-month-old are not obvious.

The differences between the topological structures of *W* ^2^, *W* ^6^, and *W* ^10^ stem from the fact that the expression of most genes has been affected by the Huntington disease as time goes on. The big differences between phase-specific gene coexpression networks indicate that the analysis of differentially coexpressed gene according to the changes of the topological structures of different networks may be helpful for understanding the changes of interactions between genes as the disease gets worse.

### 3.3. Performance Analysis of DCGN

According to Definition 2, we denoted the identification of differentially coexpressed genes based on the topological structure similarity by using (3) as DCGN-S. According to Definition 3, we denoted the identification of differentially coexpressed genes based on the variation of topological information by using (7) as DCGN-I. There is a parameter *k*, the level of the neighboring nodes, which needs to be preset in practice. In our paper, we set *k* = 1, *k* = 2, and *k* = 3 to test the performance of DCGN with different measures to evaluate the value of gene differential coexpression, including DCGN-S and DCGN-I. The following criteria were used to evaluate the identification accuracy of disease-related genes: the true positive rate (TPR), which is defined as the ratio of correctly predicted disease genes to all disease genes, and the false positive rate (FPR), which is defined as the ratio of incorrectly predicted disease genes to all nondisease genes. The receiver operating characteristic (ROC) curve was created by plotting TPR versus FPR. The area under the curve (AUC) [28] was also used as a measure of the identification accuracy.

**Figure 5:**
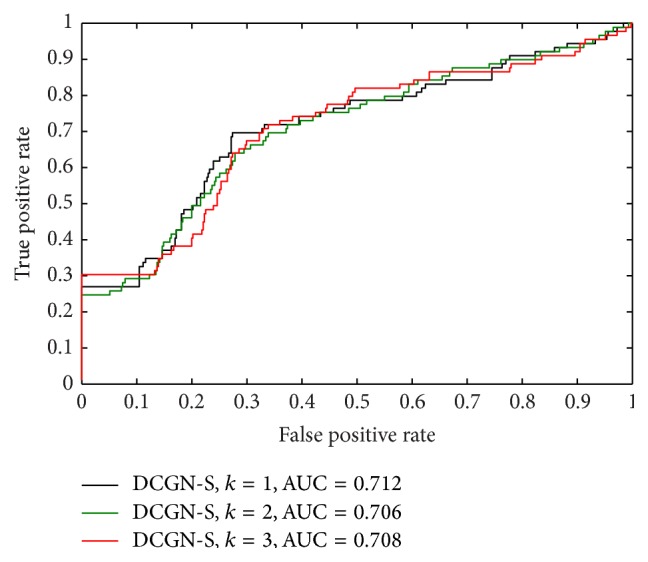
The ROC curves of DCGN-S with different *k*.

**Figure 6:**
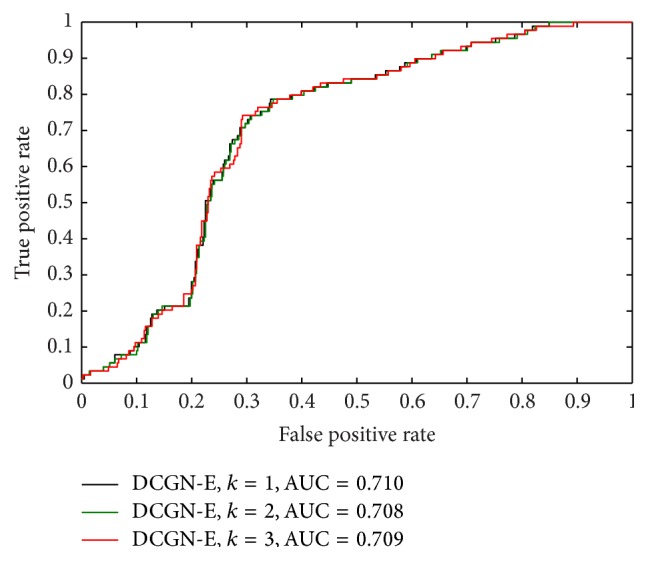
The ROC curves of DCGN-I with different *k*.

As illustrated in Figure 5, with different *k* (the level of neighboring nodes), the ROC curves of DCGN-S with different *k* are approximate. From Figure 6, it is clear that the ROC curves of DCGN-I with different *k* are also approximate. These results suggest that the performances of DCGN-S and DCGN-I are insensitive to *k*. From Table 3, it can be seen that the AUCs of DCGN-S and DCGN-I with *k* = 1 are better than *k* ≥ 2. This indicates that we may introduce redundancy information when *k* ≥ 2. Thus the performances of DCGN-S and DCGN-I get poor when *k* ≥ 2. In addition, this also increases the computational complexity of DCGN when *k* ≥ 2. Therefore, we suggest to use *k* = 1 in other experiments.

Comparing Figure 5 with Figure 6, it can be clearly seen that the ROC curves of DCGN-S are greatly different from DCGN-I. As illustrated in Figure 5, DCGN-S can distinguish disease genes from nondisease genes accurately for high ranking genes. We checked the rank lists and found that these high ranking genes share the same ranking in rank list, which means the values of differential coexpression of these genes are equal. It suggests that DCGN using the topological structure similarity can not precisely reflect the dynamic changes of the interactions between genes. As shown in Figure 6, though DCGN-I can hardly distinguish disease genes from nondisease genes for high ranking genes, the accuracy is greatly improved when FPR in [0.2,0.4]. Though the nodes with large degrees are prone to get a higher rank by using DCGN-I (the analysis is shown in Section 3.6), DCGN-I fails to accurately distinguish disease genes from nondisease genes for high ranking genes. This suggests that there is no strong and significant correlation between hub nodes and disease genes. However, the ratio of disease genes to nondisease genes in training set is approximate 1 : 5. The ratio of TPR to FPR in hub nodes (high ranking genes) is approximate 1 : 1. It demonstrates that the hub nodes are more likely to be disease genes.

### 3.4. The Performance Comparison of DCGN, RP-FC, and RP-*t*

**Figure 7:**
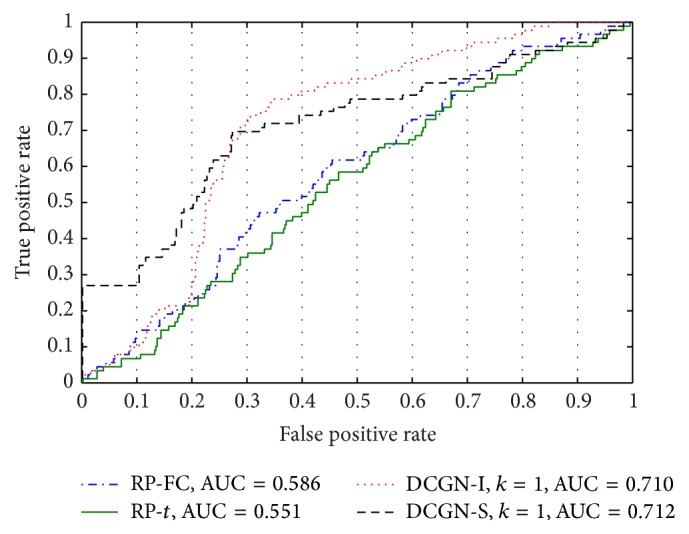
Performance comparison between RP-FC, RP-*t*, DCGN-S, and DCGN-I.

To illustrate the effectiveness of our methods, we compared it with a rank-product method based on fold-change criteria [22], denoted as RP-FC, and a rank-product model based on *t*-test, denoted as RP-*t* [4]. The comparison of ROC curves of DCGN-S, DCGN-I, RP-FC, and RP-*t* is shown in Figure 7. We also investigated the differentially coexpressed genes obtained by using DCGN and the differentially expressed genes obtained by using RP-FC and RP-*t*, and the comparison results are shown in Tables 4 and 5. We used the following criteria to compare the results of different methods and to evaluate the performance of different methods: the number of overlapped genes between different rank lists in the same ranking range, which was used to test the robustness of the results, and the percentage of overlap, which is defined as the ratio of the number of overlapped genes in the same ranking range to the length of the ranking range.

As illustrated in Figure 7, the AUCs of DCGN, including DCGN-S and DCGN-I, are far better than RP-FC and RP-*t*. From Table 3, it can be known that the AUC of DCGN-I with *k* = 1 is improved by more than 21.2% compared with the AUC of RP-FC. This indicates that the accuracy of the differentially coexpressed disease genes obtained by using DCGN is much better than that of differentially expressed genes obtained by using RP-FC and RP-*t*. Thus, the results of experiments verify the effectiveness of DCGN.

As illustrated in Table 4, when identifying differential coexpression genes by using the measure of the variation of topological information, the degree of overlap between different rank lists in the same ranking range is more than 80%, which is much higher compared to the results by other methods. It suggests that the result of DCGN-I is robust. When identifying differential coexpression genes by using the measure of topological structure similarity, the degree of overlap between different rank lists in the same ranking range is poor. It indicates that the rank of gene differential coexpression is greatly affected by parameter *k*. However, the fluctuation of the ranking of a gene is mostly controlled within 500. When identifying differentially expressed genes by using RP-FC and RP-*t*, respectively, the degree of overlap between the two rank lists in the same ranking range is very poor. It indicates the poor robustness of differentially expressed genes obtained by different methods.

We conducted further analysis of the overlapped genes in the same ranking range. As shown in Table 5, the degree of overlap between the differentially coexpressed genes obtained by using DCGN-S and the differentially coexpressed genes obtained by using DCGN-I is very poor. It illustrates that there exist big differences between the differentially coexpressed genes by using different measures to evaluate the value of gene differential coexpression. The degree of overlap between the differentially coexpressed genes obtained by using DCGN-S or DCGN-I and the differentially expressed genes obtained by using RP-FC and RP-*t* is also very poor. It suggests that there exist big differences between the differentially expressed genes which derived from the changes of gene expression levels and the differentially coexpressed genes which derived from the changes of gene coexpression networks.

### 3.5. Results in Type 2 Diabetes Mellitus Gene Expression Data

To test the feasibility and effectiveness of DCGN, we conducted experiment by using another time-series gene expression dataset of type 2 diabetes mellitus (T2DM) [29, 30]. The gene expression data was obtained from the Gene Expression Omnibus database (GSE 13271) of National Center for Biotechnology Information (NCBI). It was obtained from the white adipose tissue of disease rats aged from 4 weeks to 20 weeks, and the time interval was 4 weeks. There are 5 samples in each time point. In order to filter out noise genes, we conducted a preprocessing step and selected 5555 genes from the total 31099 genes in the gene expression data.

The T2DM related genes were downloaded from http://rgd.mcw.edu/wg/home. Totally, 202 disease-related genes were used, which were part of gene expression data in our experiment.

It is important to be noted that as there are only 5 samples in every time point, we improved the threshold to filter out large amount of false positive connections in the construction process of phase-specific gene coexpression networks. The topological information of the networks is shown in Table 6. The similarity of topological structures between any two networks is shown in Table 7.

**Figure 8:**
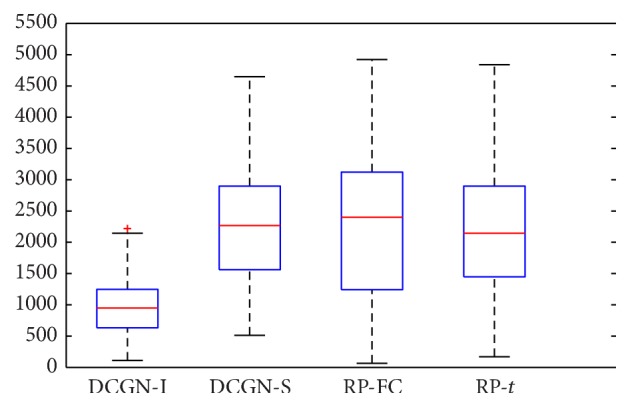
Boxplot of the rankings of disease-related genes.

Two rank lists of gene differential coexpressions were obtained by conducting gene differential coexpression analysis based on DCGN-I and DCGN-S with *k* = 1, respectively. Two rank lists of gene differential expression were obtained by conducting gene differential expression analysis based on RP-FC and RP-*t*, respectively. It needs to be noted that the high ranking of a gene in the rank list represents high probability of being a disease-related gene. We analyzed the distribution of rankings of disease-related genes in the training set, and the results are shown in Figure 8. From Figure 8, it can be seen that the average ranking of disease genes in training set by using DCGN-I is much higher than that by using other methods. From Table 5, the average degree of each gene coexpression network is in the range of [190,310], indicating that there are dense connections in those networks. For networks with such feature, DCGN-I is more suitable than DCGN-S for analyzing those networks (the analysis is shown in Section 3.6). Experimental results also show that the effectiveness of DCGN-I is much better compared to DCGN-S.

In total, by analyzing local topological structures of gene coexpression networks, DCGN-I can screen differentially coexpressed genes. Compared with traditional differentially expressed gene identification methods, DCGN-I can effectively improve the accuracy of disease-related genes selection.

### 3.6. A Case Study

**Figure 9:**
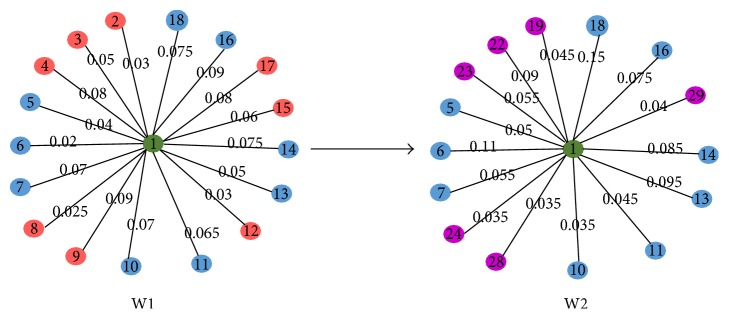
The topological structure changes of a node with large degree from W1 to W2.

**Figure 10:**
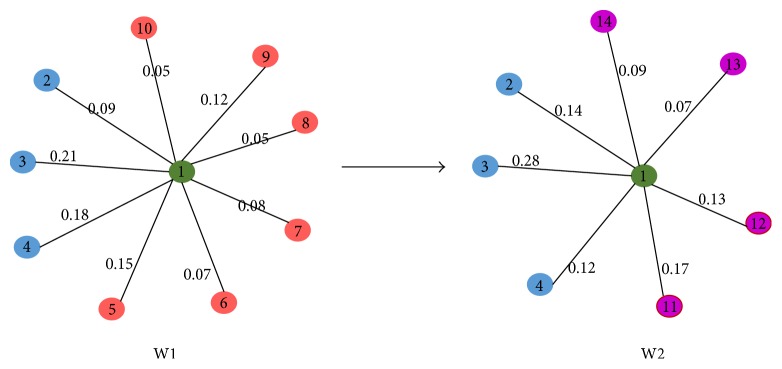
The topological structure changes of a node with small degree from W1 to W2.

In order to analyze the characters of gene differential coexpression by using different measures, we conducted a case analysis in this subsection.  Figure 9 illustrated the topological structure changes of a node, which has a larger degree in comparison with the node in Figure 10, from network W1 to network W2. Figure 10 showed the topological structure changes of a node with small degree from network W1 to network W2.

By using DCGN-S, we obtained that the value of differential coexpression of node 1 in Figure 9 is *S*_1_^12^ = 0.719 and the value of differential coexpression of node 1′ in Figure 9 is *S*_1′_^12^ = 0.813. *S*_1_^12^ < *S*_1′_^12^ means that node 1′ in Figure 10 is of larger probability of being differentially coexpressed disease gene compared to node 1 in Figure 9.

By using DCGN-I, we obtained that the value of differential coexpression of node 1 in Figure 9 is *I*_1_^12^ = 998.6 and the value of differential coexpression of node 1′ in Figure 10 is *I*_1_^12^ = 310.6. *I*_1_^12^ > *I*_1′_^12^ means that node 1 in Figure 9 is of larger probability of being differentially coexpressed disease gene compared to node 1′ in Figure 10.

In gene coexpression network *W* ^*t*^, if the degree of node *i* is large, the probability of transition from node *i* to its most neighboring nodes will be getting small. This is because ∑_*j*∈*N*_*i*_^*tk*^_ *p*_*ij*_ = 1. Since the information of an edge (see (6)), which is used to evaluate the information of a connection between nodes, is a monotone decreasing function, thus the changes of connections between large degree nodes could generate a greater value. Therefore, the identification of differentially coexpressed genes based on the variation of topological information is prone to give nodes with large degree (e.g., hub nodes) larger differential coexpression values. So, we can conclude that the nodes with significant network property of hub nodes are more likely to be screened as differentially coexpressed disease-related genes by using DCGN-I. The above characters of DCGN-I may contribute to improving the identification accuracy of disease genes [31]. From the above, the identification of differentially coexpressed genes based on the variation of topological information is more suitable for disease gene analysis of highly connected network.

From the case study it can also be seen that, for nodes with small degree, slight differences in two networks may generate large differential coexpression value when screening differentially coexpressed genes by using the measure of topological structure similarity, while, for nodes with large degree, great differences in two networks only generate small differential coexpression value. The above characters of DCGN-S may result in low accuracy of the identification of disease genes. It can be concluded that the identification of differentially coexpressed genes based on the topological structure similarity is more suitable for gene differential coexpression analysis of sparsely connected network.

In brief, the DCGN can effectively improve the accuracy of disease gene selection, while there exist large differences between the selected differentially coexpressed genes by using different measures to evaluate the value of gene differential coexpression. From the above analysis, it is also clear that DCGN-S and DCGN-I can be used to analyze networks with different topological structures.

## 4. Conclusion

Existing disease gene prediction methods mostly focus on cancer diagnosis and classification. For complex diseases with complex etiology, such as neurodegenerative diseases and diabetes mellitus, it is hard to find disease-related genes by traditional computing methods, making it difficult to discover and understand the development mechanism of these diseases.

In this paper, we designed a novel framework to identify disease-related genes and developed a differential coexpression analysis method by using time-series gene expression data. Compared with traditional analysis methods for differential expression disease-related genes, the effectiveness of DCGN for differential coexpression disease-related genes is verified.

It is reported that there usually exist a lot of false connections in gene coexpression network; thus the simulation results of coexpression network may have a great departure from real situation [32]. Therefore, constructing gene networks which accurately reflect the interactions between genes will greatly improve the performance of DCGN. In addition, the robustness of differentially coexpressed genes may be improved by integrating other information, such as weights of edges or the properties information of nodes, owing to the low percentage of overlap between the differentially coexpressed genes obtained by using DCGN-S and the differentially coexpressed genes obtained by using DCGN-I. As the percentage of overlap between the differentially coexpressed genes obtained by using DCGN and the differentially expressed genes obtained by using RP-FC and RP-*t* is poor, the identification accuracy of disease genes may be greatly improved by integrating the differential expression information of nodes into the process of differential coexpression analysis. We will conduct relevant studies about the strategies mentioned above.

## Figures and Tables

**Table 1:** RNA-seq data of Huntington disease mice.

Tissue	Genotype	Age
Striatum	polyQ 92	2-month-old 6-month-old 10-month-old
	polyQ 111	
	polyQ 140	
	polyQ 175	

**Table 2:** Topological information of gene coexpression networks.

Network	*W* ^2^	*W* ^6^	*W* ^10^
Nodes number	8815	8815	8815
Edges number	7797404	1433744	628150
Average degree	1024.09	168.08	89.58
Average weight	0.423	0.339	0.336
Scatters number	1201	285	803

**Table 3:** The AUC of each experiment.

Method	*k* = 1	*k* = 2	*k* = 3
MFSN-S	**0.7118**	0.7062	0.7083
MFSN-I	**0.7101**	0.7081	0.7094

RP-FC	0.5856		
RP-*t*	0.5513		

*Note.* Bold indicates the best values.

**Table 4:** The number of overlapped genes (the degree of overlap) between different rank lists in the same ranking range.

Ranking range	DCGN-S	DCGN-I	RP-FC ∩ RP-*t*
Unit: 10^3^	*k* = 1,2, 3	*k* = 1,2, 3	—
[0,1]	489 (0.49)	**926 (0.93)**	325 (0.33)
[1,2]	153 (0.15)	**830 (0.83)**	186 (0.19)
[2,3]	118 (0.12)	**809 (0.81)**	169 (0.17)
[3,4]	86 (0.09)	**830 (0.83)**	224 (0.22)
[0,2]	1269 (0.63)	**1896 (0.95) **	991 (0.50)
[0,3]	2242 (0.75)	**2904 (0.97)**	1913 (0.64)
[0,4]	3152 (0.79)	**3919 (0.98)**	2768 (0.69)

**Table 5:** The number of overlapped genes (the degree of overlap) by using different methods.

Ranking range	DCGN-S	DCGN-S	DCGN-I
Unit: 10^3^	∩ DCGN-I	∩ RP-FC ∩ RP-*t*	∩ RP-FC ∩ RP-*t*
[0,1]	1 (0.001)	4 (0.004)	**13 (0.013)**
[0,2]	5 (0.003)	48 (0.024)	**78 (0.039)**
[0,3]	164 (0.055)	232 (0.077)	**278 (0.093)**
[0,4]	509 (0.013)	637 (0.016)	**743 (0.019)**

**Table 6:** Topological information of spatial-specific gene coexpression networks for T2DM.

Network	*W* ^4^	*W* ^8^	*W* ^12^	*W* ^16^	*W* ^20^
Nodes number	5555	5555	5555	5555	5555
Edges number	1712916	1656238	1312428	1167228	1104506
Ave degree	308.4	298.2	236.3	210.1	198.8
Ave weight	0.660	0.655	0.650	0.646	0.644
Scatters number	0	0	0	0	0

**Table 7:** The similarity of topological structures between any two phase-specific gene coexpression networks for T2DM.

Network	*W* ^8^	*W* ^12^	*W* ^16^	*W* ^20^
*W* ^4^	0.029	0.037	0.037	0.034
*W* ^8^		0.035	0.029	0.025
*W* ^12^			0.031	0.036
*W* ^16^				0.030
